# Characterizing a large outbreak of dengue fever in Guangdong Province, China

**DOI:** 10.1186/s40249-016-0131-z

**Published:** 2016-05-03

**Authors:** Jian-Peng Xiao, Jian-Feng He, Ai-Ping Deng, Hua-Liang Lin, Tie Song, Zhi-Qiang Peng, Xiao-Cheng Wu, Tao Liu, Zhi-Hao Li, Shannon Rutherford, Wei-Lin Zeng, Xing Li, Wen-Jun Ma, Yong-Hui Zhang

**Affiliations:** Guangdong Provincial Institute of Public Health, Guangdong Provincial Center for Disease Control and Prevention, Guangzhou, China; Guangdong Provincial Center for Disease Control and Prevention, Guangzhou, China; School of Public Health, Sun Yat-sen University, Guangzhou, China; Center for Environment and Population Health, Griffith University, Brisbane, Australia

**Keywords:** Dengue fever, Epidemiology, Outbreak, China

## Abstract

**Background:**

Dengue cases have been reported each year for the past 25 years in Guangdong Province, China with a recorded historical peak in 2014. This study aims to describe the epidemiological characteristics of this large outbreak in order to better understand its epidemic factors and to inform control strategies.

**Methods:**

Data for clinically diagnosed and laboratory-confirmed dengue fever cases in 2014 were extracted from the China Notifiable Infectious Disease Reporting System. We analyzed the incidence and characteristics of imported and indigenous cases in terms of population, temporal and spatial distributions.

**Results:**

A total of 45 224 dengue fever cases and 6 deaths were notified in Guangdong Province in 2014, with an incidence of 47.3 per 100 000 people. The elderly (65+ years) represented 11.7 % of total indigenous cases with the highest incidence (72.3 per 100 000). Household workers and the unemployed accounted for 23.1 % of indigenous cases. The majority of indigenous cases occurred in the 37^th^ to 44^th^ week of 2014 (September and October) and almost all (20 of 21) prefecture-level cities in Guangdong were affected. Compared to the non-Pearl River Delta Region, the Pearl River Delta Region accounted for the majority of dengue cases and reported cases earlier in 2014. Dengue virus serotypes 1 (DENV-1), 2 (DENV-2) and 3 (DENV-3) were detected and DENV-1 was predominant (88.4 %).

**Conclusions:**

Dengue fever is a serious public health problem and is emerging as a continuous threat in Guangdong Province. There is an urgent need to enhance dengue surveillance and control, especially for the high-risk populations in high-risk areas.

**Electronic supplementary material:**

The online version of this article (doi:10.1186/s40249-016-0131-z) contains supplementary material, which is available to authorized users.

## Multilingual abstracts

Please see Additional file 1 for translations of the abstract into the five official working languages of the United Nations.

## Background

Dengue is an arthropod-borne viral disease caused by any of four dengue virus serotypes (DENV 1–4) and *Aedes* mosquitoes serve as the main transmission vector of this infection [1]. Currently about half of the global population lives in areas at risk of dengue infection and dengue is regarded as the most prevalent mosquito-borne viral disease in humans. It has been estimated that 390 million people have had dengue virus infection in more than 100 countries, and dengue incidence has increased 30-fold in the past 50 years [1].

Dengue is not a new infectious disease in China [2]. After the 1970s there were several large outbreaks of dengue fever in southern China (Hainan, Guangxi, Fujian, Zhejiang, Yunnan and Guangdong Province). All of these provinces are located in the southeast coastal region or around national borders with Myanmar, Laos and Vietnam [3]. From the 1990s to 2010s, dengue in China was characterized by sporadic outbreaks or imported cases [4]. However, dengue incidence has increased since 2012, peaking in 2014 [5, 6], and a total of 655 324 cases of dengue were reported in mainland China in 2014 and populations aged 20-49 years and those unemployed and retired were the most heavily affected [6].

Guangdong Province is located in southern China. It has a hot and humid sub-tropical climate and being adjacent to the Hong Kong and Macao Special Administrative Region. Guangdong has frequent economic and cultural communication with Southeast Asia where dengue is endemic [7, 8]. It is a highly developed region of China with densely populated urban cities, such as Guangzhou, Foshan and Shenzhen, which are in the heart of the Pearl River Delta. Guangdong Province has been one province most seriously affected by dengue in mainland China, and the prevention and control of dengue has been a high priority in this province since its emergence in Foshan City in 1978 [9]. Dengue cases have been reported each year in Guangdong from 2005 to 2014, increasing rapidly in the last three years [5]. In 2014, the number of dengue cases was the highest on record constituting the largest dengue epidemic in Guangdong in the past 25 years [5]. It is very important to understand the epidemiological characteristics of this large outbreak and extract lessons about how it could be averted in the future.

In this study we described the population, temporal and geographical distribution of dengue cases in 2014 using data from the China Notifiable Infectious Disease Reporting System, with the aim of identifying the drivers of the large outbreak, thereby helping to better refine existing dengue prevention strategies.

## Methods

### Data collection

Dengue fever was classified as a Class B notifiable infectious disease in China in 1989 [10]. Within 24 h following a dengue diagnosis, the disease must be reported by doctors to the online National Notifiable Infectious Disease Reporting Information System (NNIDRIS) at the Chinese Center for Disease Control and Prevention. Information recorded for each case includes gender, age, address, nationality, type of diagnosis (suspected, clinical, laboratory), virus serotype, imported or indigenous case, date of illness onset, and potential risk factors. Dengue cases are diagnosed according to the unified diagnosis criteria issued by the Chinese Ministry of Health.

Daily dengue data in 2014 in Guangdong Province were obtained from the NNIDRIS. All the case data used in this study was anonymous. The surveillance was determined by the Chinese Ministry of Health to be a part of continuing public health surveillance and was exempted from institutional review board assessment [5].

Dengue cases are generally classified as probable or confirmed based on whether they are clinically diagnosed or laboratory confirmed. Clinically diagnosed cases and laboratory confirmed cases were included in data used for the current research. An imported case of dengue is defined as a dengue case for which the patient had traveled to a dengue-affected foreign country or province of mainland China, and reported being bitten by mosquitoes within 15 days of the onset of illness [11]. In the present study, imported cases were only from foreign countries. A determination of whether a case was imported or indigenous was made by local public health institutes, following epidemiological investigations.

### Data analysis

The crude incidence rate was calculated as the number of dengue cases divided by the population. The epidemiologic characteristics of indigenous and imported cases in Guangdong in 2014 were summarized by age, gender and occupation.

To identify high-risk areas of dengue fever, we plotted the geographical distribution of cases by town (there were 1903 towns in Guangdong in 2014), and then used spatial scan statistics to analyze the spatial cluster for dengue fever [12]. To describe the seasonal patterns of dengue cases in different cities, we created heat maps of the mean value of the proportion of cases for each city in each week. The weekly cases were standardized by the maximum number of cases per week, and classified by whether they were in the Pearl River Delta (PRD) or non- Pearl River Delta (nPRD) region.

We used R software version 3.0.0 (R Development Core Team 2013) to produce the graphs and heat maps and conduct statistical analyses. ArcGIS 10.0 (ESRI, Redlands, CA, USA) was used to plot the geographical distribution. SaTScan 9.4 was used to analyze spatial clusters.

## Results

### Population distribution

In 2014, a total of 45 224 dengue fever cases including 6 deaths were reported in Guangdong Province. Of them, 45 123 (99.8 %) cases were indigenous and 101 (0.2 %) were imported cases. The incidence rate of dengue was 42.5 per 100 000 population.

There was a similar gender distribution for indigenous cases though there were more males (1.24:1) among imported cases. The age distribution differed between imported and indigenous cases, with an older median age of 40.0 years (IQR: 26.0-54.0) for indigenous cases and a younger median age of 32.0 years (IQR: 25.5-45.0) for imported cases. Those aged 65 years and over had the highest incidence (72.3 per 100 000) compared with other age groups. Household workers and the unemployed represented the largest groups for both indigenous and imported cases, followed by cases who were retired or working in business services (Table 1).

**Table 1:** Numbers of dengue fever cases by gender, age and employment status in Guangdong in 2014

Variable	Indigenous cases (Incidence rate/10^5^)	Imported cases (Incidence rate/10^5^)	Total (Incidence rate/10^5^)
Total cases	45123 (42.4)	101 (0.1)	45224 (42.5)
Gender			
Male	22419 (40.4)	56 (0.1)	22475 (40.5)
Female	22704 (44.6)	45 (0.1)	22749 (44.7)
Gender ratio	0.99	1.24	0.99
Age Group (years)			
< 5	925 (14.6)	0 (0)	925 (14.6)
5-14	2284 (21.1)	3 (0.03)	2287 (21.2)
15-24	6569 (29.3)	17 (0.1)	6586 (29.4)
25-34	9717 (48.8)	32 (0.2)	9749 (49.0)
35-44	7999 (44.9)	23 (0.1)	8022 (45.0)
45-54	6759 (50.7)	14 (0.1)	6773 (50.8)
55-64	5581 (66.1)	6 (0.1)	5587 (66.2)
≥65	5289 (72.3)	6 (0.1)	5295 (72.4)
Employment status (constituent ratio %)			
Household/unemployed	10439 (23.1)	33 (32.7)	10472 (23.1)
Retired	5874 (13.0)	16 (15.8)	5890 (13.0)
Business service	5712 (12.7)	11 (10.9)	5723 (12.7)
Worker	4915 (10.9)	8 (7.9)	4923 (10.9)
Student	3354 (7.4)	6 (5.9)	3360 (7.4)
Farmer	2182 (4.8)	7 (6.9)	2189 (4.8)
Administrative staff	1697 (3.8)	2 (2.0)	1699 (3.8)
Other	2919 (6.5)	7 (6.9)	2926 (6.5)
Unknown	8031 (17.8)	11 (10.9)	8042 (17.8)

### Temporal distribution

**Fig. 1:**
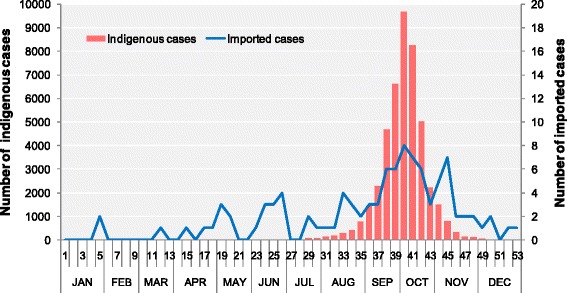
Temporal distribution of indigenous and imported dengue cases in Guangdong Province, China in 2014

Figure 1 shows the temporal distribution of cases throughout 2014. The imported cases of dengue fever were scattered throughout the year. However, the indigenous cases were first reported in the 24^th^ week, with rapid increases in the following 15 weeks and a peak in the 40^th^ week (coinciding with the National Day in this week). After the 40^th^ week, the number of cases reduced significantly. The peak of the epidemic was observed in September and October 2014.

### Spatial distribution

**Fig. 2:**
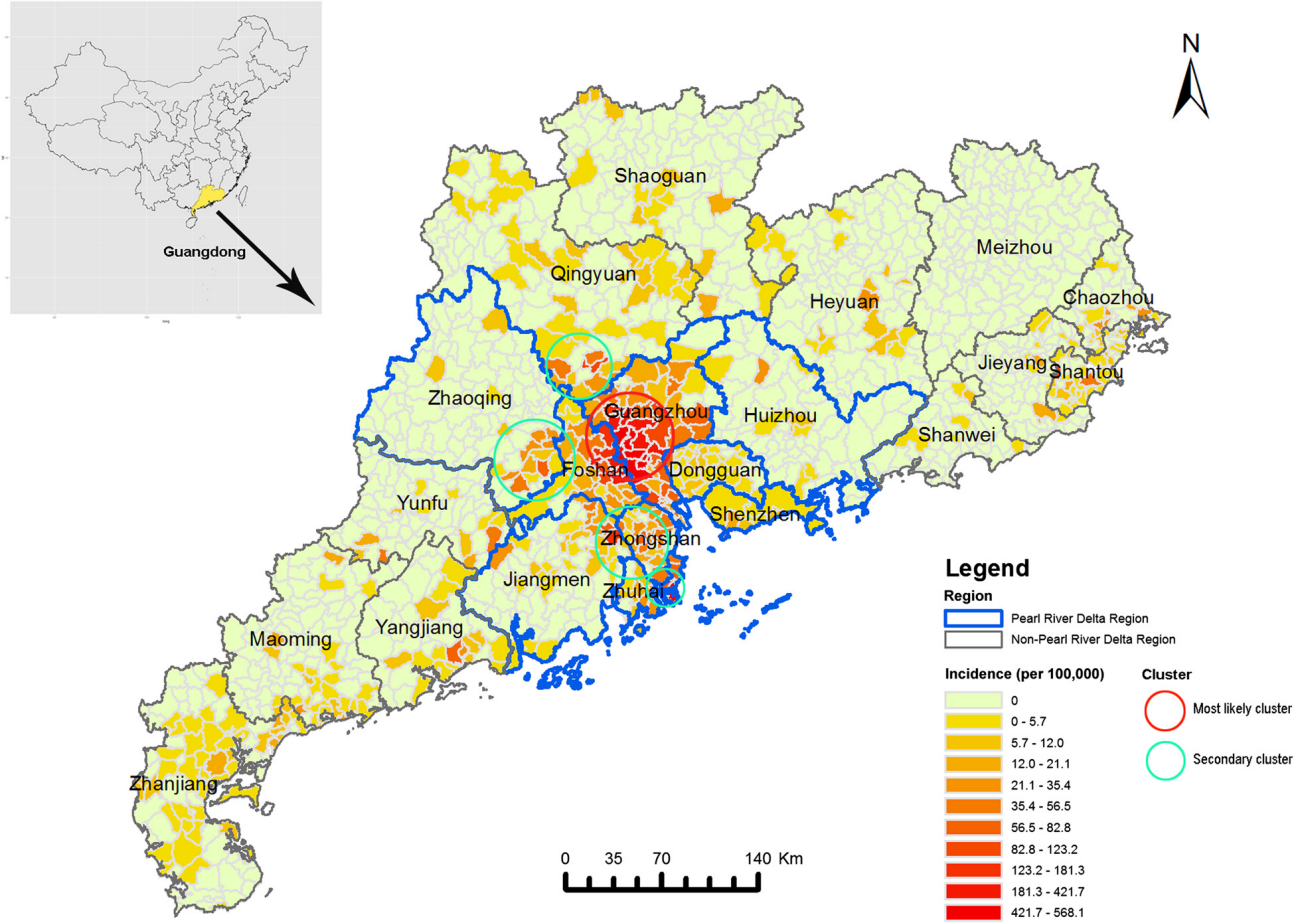
Geographical distribution of dengue fever in Guangdong Province, China in 2014

All prefecture-level cities except for Meizhou City in Guangdong Province reported dengue cases in 2014 (Fig. 2). The spatial clusters analysis revealed five spatial clusters, including one most likely cluster and four secondary clusters. The most likely cluster (depicted by the red circle in Fig. 2) consisted of 90.5 % of cases and was located in central Guangdong. The cluster covered two cities, Guangzhou and Foshan, which are the most economically developed and densely populated cities in the PRD. Guangzhou, the capital of Guangdong Province, had the highest case number (37 382 cases), accounting for 82.7 % of all cases. The secondary clusters (depicted by green circles in Fig. 2) were located in Zhongshan, Jiangmen, Zhuhai, Zhaoqing and Qingyuan. These cities are near to Guangzhou and Foshan.

### Tempo-spatial distribution

**Fig. 3:**
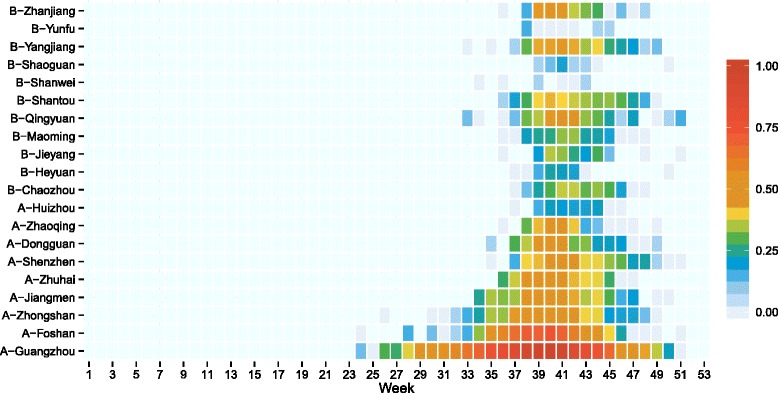
Heat map of indigenous dengue cases by city and week in Guangdong Province in 2014. Those cities labeled with an A are in the Pearl River Delta Region. Those with a B label are outside the Pearl River Delta Region. The number of weekly cases was standardized by the number of total cases

Figure 3 further depicts the tempo-spatial distribution of indigenous dengue fever cases in 2014. We found that indigenous cases were first reported in Guangzhou in the 24^th^ week, followed by Foshan and Zhongshan, which are geographically close to each other. From the 37^th^ week, dengue fever cases were reported in all cities in the PRD. Cases were observed outside of the PRD from the 37^th^ week. Qingyuan, near to Guangzhou, had the most cases in the nPRD. Imported cases were mainly reported in the cities of the PRD, such as Guangzhou and Shenzhen (Additional file 2: Figure S1).

### Virology

In 2014, 48.5 % (21 940/45 224) of the reported dengue cases were laboratory confirmed. Data on serotypes were available for 345 (0.8 %) of indigenous cases, of which, 305 (88.4 %) cases were serotype DENV-1 and 40 cases (11.6 %) were serotype DENV-2. Only two (2.0 %) imported cases’ serotype were available and they were DENV-1 and DENV-3, respectively.

## Discussion

The incidence of dengue fever has increased markedly worldwide in recent decades due to globalization, urbanization, climate change and other factors [1, 6, 13]. In 2014, Guangdong Province experienced a large dengue outbreak, the largest dengue epidemic in the past 25 years. Although it is not clear what was responsible for this outbreak, it is consistent with the increasing trend seen around the globe [4]. In recent years, the incidence of dengue in China’s neighboring countries like Malaysia, Singapore and Indonesia has been higher compared to previous years [14, 15]. In 2014, adjacent regions such as Hong Kong and Taiwan have also seen increased cases compared to 2013 [16]. Guangdong Province has established close exchanges with these regions due to globalization. Hence imported dengue virus associated with returning Chinese travelers or visitors from these regions is expected.

Weather factors may also played an important role in the large outbreak in 2014. The Guangdong meteorological service showed that the average monthly temperatures in Guangdong from June to September were 0.1-1.3 °C higher in 2014 than in previous years and the rainfall was 63 % higher than usual [17]. Recent studies found that the increases of temperature and rainfall may result in increased survival of *Aedes* mosquitoes and the potential for dengue transmission in Guangdong [18, 19]. The high level of urbanization in the PRD may also be an important risk factor for dengue epidemic and transmission. The PRD has experienced remarkable urban expansion over the past three decades: 32.8 % of the land area has been converted into urban use during the period from 1992 to 2012 [20, 21]. One study found that urbanization in Guangzhou had increased *Aedes albopictu*s (the main dengue vector in Guangdong Province) larval habitats and accelerated mosquito development and survivorship [22]. In addition, the dense population in the highly urbanized areas may further promote dengue transmission [13].

The current study found that the elderly (65+ years) had the highest incidence in 2014, and 5 of the 6 deaths were the elderly. This result was consistent with a previous study in Taiwan region which indicates the elderly are at particularly high risk for dengue virus–related sickness and death [23]. There are two possible explanations for this. Firstly, the elderly are more likely to spend more time outdoors compared with younger people in summer, and hence vector exposure may be substantially higher in this age group [23]. Secondly, the elderly are more likely to have existing chronic diseases that are identified as possible risk factors for severe dengue fever. This finding is different from that reported in India and Southeast Asia where most dengue cases are children or younger adults [24–26]. This pattern is likely due to the fact that dengue is not an endemic disease in Guangdong Province and hence the population has very low seroprevalence of dengue antibodies making them broadly susceptible to dengue infection. Whereas the populations in dengue endemic countries have higher rates of immunity, especially in adults and the elderly [9]. Therefore, the elderly are at high risk from dengue infection in Guangdong and may be at risk of severe clinical outcomes.

In terms of occupations, household workers and the unemployed represented the major proportion of dengue cases. This result is consistent with a previous report in Colombia where a high proportion of cases were either unemployed or those working at home [27]. Since dengue fever is transmitted by the *Aedes* mosquito, and the *Aedes* mosquitoes breed in clear, collected water, household workers and the unemployed tend to spend more time at home or in the community where there are more breeding places for *Aedes* mosquitoes [28]. Research in Guangdong found that household workers liked walking in parks, going to the food market, or raising aquatic plants, and as these areas were all potential mosquito breeding grounds, they were at risk of higher vector exposure [9]. Moreover, the groups often have lower levels of education and limited protective knowledge [29]. These findings implied that improvements in dengue prevention strategies for household workers and the unemployed should be considered in the future control practice.

Through the spatial cluster analysis, we found the epidemic center of the 2014 event was in the PRD. The PRD is a densely populated region and a major transit center with a high level of urbanization [22]. This finding is consistent with previous reports that clusters are more commonly observed in the areas of high population density and mobility [30, 31]. In addition, the poor solid waste management in some villages of the PRD is common, which may contribute to the outbreak of dengue fever [32]. Heat maps demonstrated that Guangzhou was first hit by dengue fever, followed by the neighboring cities. This pattern may relate to the rapid transit systems and close trading ties between these cities [33]. Before the 37^th^ week, the dengue epidemic was mainly limited to the PRD, after that two national festivals (the Mid-Autumn Festival and National Day Holiday) promoted population movement from PRD cities to those cities outside the PRD region as migrants travelled back to their home town in the festivals [34]. Previous studies in Cambodia, Thailand and Vietnam have reported similar results: population movement plays a major role in the regional spread of dengue fever [35–37]. With the rapid growth of the economy and urbanization in Guangdong, more and more people have moved away from rural areas to urban cities in the PRD, increasing city size and industry and hence contributing to increased risk of dengue fever. Therefore, further exploring the role of human movement on the dengue transmission in Guangdong is significant, which would provide valuable information for planning dengue prevention and outbreak response in the future.

In our study, three serotypes of dengue virus (DENV-1, DENV-2 and DENV-3) were found in the dengue outbreak in 2014, with DENV-1 being the dominant serotype. DENV-1 has been the most predominant in most years in Guangdong [9], with the other three serotypes, DENV-2, DENV-3, and DENV-4 only occasionally isolated in the past decade [38]. Previous studies showed that the dengue virus strains from each year in Guangdong belonged to different genotypes [39]. However, analysis of the newly isolated DENV-2 in Guangzhou during the 2014 outbreak demonstrated high homology to the ones isolated in the past decade in Guangdong [40]. The high homology and identical genotype of Guangdong DENV-2 strains suggest the possibility of establishment of local DENV-2 infection in Guangdong [40]. It is important to establish virological surveillance to clearly understand the current dengue epidemic and virological characteristics of dengue virus in Guangdong. Dengue antibodies only prevent re-infection by the same serotype and when people experience a secondary dengue infection with a new serotype they face a much greater risk of developing severe dengue indicating that pre-existing antibodies to DENV can exacerbate disease [41, 42]. Following many years of dengue outbreaks, Guangdong now has a large number of the population with antibodies to DENV-1. This will be good for future protection against DENV-1, but it implies that quite a large population are now at risk of severe dengue if there are new or different serotypes circulating, and children and the elderly with previous infections would be the most-at-risk populations. 321 cases (0.7 %) were identified as severe in 2014 [43], and this proportion may rise if other large outbreaks occur in future years with changing serotype prevalence. Hence, sero-epidemiological surveillance in the early stages of a dengue epidemic is crucial for designing health education and promotion programs, particularly for targeting the highest risk populations and for correct messaging about signs and symptoms of the severe forms of dengue fever such as severe dengue. In particular, health care practitioners need to be targeted to be vigilant in detecting and treating the more severe forms of dengue fever. Understanding the serotypes circulating in neighbouring countries and in countries where many imported cases may originate from is also important.

### Limitations

Some limitations of this study must be mentioned. Firstly, the data used in this study were collected from the passive public health surveillance system. The data quality may be influenced by the reporting methods, under-reporting or misdiagnosis, availability of health facilities and laboratory diagnostics. Secondly, for some cases there was insufficient information collected on indigenous and imported classification and some employment status information was unclear. Thirdly, due to lack of molecular biology information available for most dengue fever cases, further study is needed to understand their phylogenetic relationships and to determine the source of infections.

## Conclusions

In 2014 Guangdong experienced the most serious dengue outbreak since the 1990s suggesting dengue being a serious public health threat in Guangdong Province. There is an urgent need for enhancing dengue surveillance and early warning to response to global expansion trend of dengue fever.

## Additional files

Additional file 1: Multilingual abstracts in the five official working languages of the United Nations. (PDF 299 kb) Additional file 2: Figure S1. Heat map of imported dengue cases by city and week in Guangdong Province in 2014. A indicates the Pearl River Delta Region; B labels the non-Pearl River Delta Region. The number of weekly cases was standardized by the number of total cases. (PDF 9 kb)

## Abbreviations

CDC: Center for Disease Control and Prevention
DENV: dengue virus
IQR: interquartile range
nPRD: non- Pearl River Delta
PRD: Pearl River Delta
