# Supplementary material for: Characterizing a large outbreak of dengue fever in Guangdong Province, China
Source: Infect Dis Poverty. 2016 May 3;5:44. doi: 10.1186/s40249-016-0131-z (PMC4853873; doi:10.1186/s40249-016-0131-z)
Supplement: Additional file 1: — Multilingual abstracts in the five official working languages of the United Nations. (PDF 299 kb) [file 40249_2016_131_MOESM1_ESM.pdf]

Translation of the abstract into the five official working languages of the United Nations

وصف تفشي مرض حمى الضنك في إقليم قوانغدونغ، الصين

جيان بينغ شياو، جيان فينغ هي، أي بينغ دينغ، هوا يانغ لي، تى سونغ، زي غيانغ بينغ، وو شاوشينغ، ليو تاو ، زهو لي، شانون روذفورد، شينغ لي، ون جون ما، يونغ هوي تشانغ

ملخص

الخلفية: يتم الإبلاغ كل عام على مدار 25 عامًا السابقة عن حالات حمى الضنك في إقليم قوانغدونغ، الصين مع ذروتة المُسجلة في عام 2014. تهدف الدراسة إلى وصف الخصائص الوبائية لهذا التفشي الهائل من أجل فهم أفضل لعواملة الوبائية وإبرغ استراتيجيات المكافحة.

الطرق: تم استخلاص معطيات الحالات المُشخصة سريريًا والمؤكدة مُختبريًا لحالات حمى الضنك عام 2014 من نظام الإبلاغ عن الأمراض التي يجب الإبلاغ عنها في الصين. قمنا بتحليل الحادثة وخصائص الحالات الواردة والمُستوطنة من السكان، والتوزيعات الزمانية والمكانية .

النتائج: تم الإبلاغ عن حالات حمى الضنك بمجموع 45.224 حالة و عدد 6 وفيات في إقليم قوانغدونغ عام 2014، مع زيادة 47.3 لكل 100.000 شخص. يمثل كبار السن (سن >= 65 عامًا) نسبة 11.7% من مجموع الحالات المحلية وكانت تمثل أعلى معدل للانتشار (72.3 كل 100.000). وبلغت نسبة الإصابه في اوساط العمال المنزليين والعاطلين عن العمل 23.1% من الحالات المحلية. حدثت غالبية الحالات المحلية في الأسبوع 37 إلى 44 عام 2014 (سبتمبر وأكتوبر) واصيبت تقريبًا (20 من 21) مدينة على مستوى المحافظة في مقاطعة قوانغدونغ. ابلغ المسؤولين في منطقة نهر اللؤلؤة على الحالات في وقت مُبكر من العام وكانت معظمها حالات حمى الضنك، مقارنة بالمدن غير الواقعة على دلتا نهر اللؤلؤة. تم اكتشاف الأنماط المصليه 1 (DENV-1), 2 (DENV-2) و 3 (DENV-3) وكانت DENV-1 الأغلبية (88.4%).

الاستنتاجات: يمثل مرض حمى الضنك مشكلة خطيرة تهدد الصحة العامة، ويبرز بوصفه تهديدًا في إقليم مقاطعة قوانغدونغ. هناك حاجة ملحة لتعزيز مراقبة حمى الضنك، لا سيما بالنسبة إلى السكان والمناطق المعرضة للخطر.

Translated from English version into Arabic by Alain Alameddine, through

TRANSLATORS
WITHOUT BORDERS

2014 年中国广东省登革热暴发流行病学特征分析

肖建鹏, 何剑峰, 邓爱萍, 林华亮, 宋铁, 彭志强, 吴晓程, 刘涛, 李志浩, Shannon Rutherford, 曾韦霖, 李杏, 马文军, 张永慧

摘要

引言: 2014 年中国广东省登革热发病人数为 25 年来之最，为了更好地了解登革热的流行因素和制定防控策略，本研究描述了此次暴发流行特征。

方法：通过国家法定传染病报告系统收集 2014 年广东省登革热临床和实验室诊断病例数据，分析登革热本地病例和输入病例的发病情况、人群特征、时间和空间分布。

结果：2014 年广东省共报告登革热病例 45224 例，其中死亡 6 例，人群发病率为 47.3/100000。老年人占总本地病例的 11.7%，发病率为各年龄组中最高，达 72.3/100000。本地病例中家务人员和失业人员比例较高，为 23.1%。本地病例高峰出现在 2014 年的第 37 周到 44 周之间（9 月和 10 月）。全省 21 个地市中有 20 个报告登革热病例，珠三角地区登革热病例比非珠三角地区出现更早、流行更严重。登革病毒 1, 2, 3 血清型均有检出，其中以登革病毒 1 型占主导（88.4%）。

结论：登革热是广东省一个严重的公共卫生问题，未来将持续威胁该省居民健康，亟需加强登革热监测，

尤其对高风险地区和高风险人群。

Translated from English version into Chinese by Xiao Jianpeng

Caractérisation d'une vaste épidémie de dengue dans la province chinoise du Guangdong

Jianpeng Xiao, Jianfeng He, Aiping Deng, Hualiang Lin, Tie Song, Zhiqiang Peng, Xiaocheng Wu, Tao Liu, Zhihao Li, Shannon Rutherford, Weilin Zeng, Xing Li, Wenjun Ma, Yonghui Zhang

Résumé

Contexte : Depuis 25 ans, des cas de dengue sont rapportés chaque année dans la province chinoise du Guangdong, avec un pic historique en 2014. Notre étude décrit les caractéristiques épidémiologiques de cette vaste épidémie, afin de mieux comprendre ses facteurs épidémiques et d'apporter des informations pour l'élaboration de stratégies de lutte.

Méthodes : Les données de cas de dengue diagnostiqués cliniquement et confirmés par les analyses de laboratoire en 2014 ont été extraites du Système de notification des maladies à déclaration obligatoire chinois. Nous avons analysé l'incidence et les caractéristiques des cas importés et indigènes en termes de répartition démographique, temporelle et spatiale.

Résultats : Au total, 45 224 cas de dengue et 6 décès ont été déclarés dans la province du Guangdong en 2014, soit une incidence de 47,3 cas pour 100 000 habitants. Les personnes âgées (65 ans et plus) représentaient 11,7 % du total des cas indigènes et la plus forte incidence (72,3 pour 100 000). Les personnes au foyer et les chômeurs représentaient 23,1 % des cas indigènes. La majeure partie de ceux-ci se sont déclarés entre la 37ème semaine et la 44ème semaine de 2014 (septembre et octobre) et presque toutes les villes de préfecture du Guangdong (20 sur 21) ont été touchées. Par rapport aux autres régions de la province, la région du Delta de la Rivière des Perles a signalé des cas plus tôt dans l'année et c'est là que la majorité des cas de dengue ont été recensés. Les sérotypes 1 (DENV-1), 2 (DENV-2) et 3 (DENV-3) du virus de la dengue ont été détectés, avec une prédominance de DENV-1 (88,4 %).

Conclusions : La dengue constitue un grave problème de santé publique et une menace permanente dans la province du Guangdong. Il est urgent de renforcer sa surveillance, en particulier pour les populations menacées et dans les zones à haut risque.

Translated from English version into French by Suzanne Assenat, through

TRANSLATORS WITHOUT BORDERS

Характеристика крупной вспышки лихорадки денге в провинции Гуандун Китая

Цзяньпэн Сяо, Цзяньфэн Хэ, Айпин Дэн, Хуалян Линь, Те Линь, Чжицян Пэн, Сяочэн У, Тао Лю, Чжихао Ли, Шеннон Рутерфорд, Вэйлинь Цзэн, Син Ли, Вэньцзюнь Ма, Юнхуэй Чжан

Реферат

Предисловие: В провинции Гуандун Китая в последние 25 лет каждый год сообщалось о случаях лихорадки денге, а исторический пик был зарегистрирован в 2014 г. Целью настоящего исследования является описание эпидемиологических характеристик этой самой большой вспышки с целью лучшего понимания ее эпидемических факторов и предоставления информации для выработки стратегий сдерживания.

Методы: Данные по клинически диагностированным и лабораторно подтвержденным случаям лихорадки денге были взяты из китайской системы оповещения о подлежащих регистрации заболеваниях. Нами проанализированы заболеваемость и характеристики привнесенных и местных случаев с точки зрения распространения среди населения и временного и пространственного распространений.

Результаты: В провинции Гуандун в 2014 г. сообщалось о 45224-х случаях и шести смертях при заболеваемости 47,3 на 100 000 человек. Пожилые (в возрасте 65 лет и старше) представляли 11,7% от общих местных случаев и проявляли наивысшую заболеваемость (72,3 на 100000). На работающих дома и безработных приходилось 23,1% от местных случаев. Большинство местных случаев произошло в период с 37-ой по 44-ю недели 2014 г. (сентябрь и октябрь), при этом были затронуты почти все (20 из 21) города префектурного уровня в провинции Гуандун. В отличие от других районов, в регионе дельты Жемчужной реки и ранее сообщалось о случаях денге, там же было и большинство случаев. Были обнаружены серотипы 1 (DENV-1), 2 (DENV-2) и 3 (DENV-3) вируса денге, при этом DENV-1 являлся преобладающим (88,4%).

Выводы: Лихорадка денге является серьезной проблемой общественного здравоохранения и превращается в постоянную угрозу в провинции Гуандун. Имеется срочная необходимость в укреплении отслеживания денге, особенно среди групп населения повышенного риска и в районах повышенного риска.

Translated from English version into Russian by Alexander Somin, through

TRANSLATORS
WITHOUT BORDERS

Gran brote de dengue en la provincia de Guangdong, en China

Jianpeng Xiao, Jianfeng He, Aiping Deng, Hualiang Lin, Tie Song, Zhiqiang Peng, Xiaocheng Wu, Tao Liu, Zhihao Li, Shannon Rutherford, Weilin Zeng, Xing Li, Wenjun Ma y Yonghui Zhang

Resumen

Contexto: Durante los últimos 25 años se han registrado casos de dengue en la provincia de Guangdong, en China, habiendo alcanzado su récord histórico en 2014. Este estudio tiene como objeto describir las características epidemiológicas de este gran brote, a fin de entender mejor sus factores epidémicos y poder informar sobre las estrategias de control.

Métodos: Se recopilaron del Sistema de Información sobre Enfermedades de Notificación Obligatoria de China todos los datos sobre casos de dengue confirmados en 2014 mediante diagnóstico clínico y análisis sanguíneos. Analizamos su incidencia y características, en términos de población y de una distribución tanto temporal como espacial, que incluyese además tanto los casos importados como los autóctonos.

Resultados: En la provincia de Guangdong en 2014, se notificaron un total de 45 224 casos de enfermedad por dengue y 6 muertes, con una incidencia del 47,3 por cada 100 000 personas. El sector de población de personas mayores (edad >=65 años) representó el 11,7 % del total de los casos autóctonos, siendo la mayor incidencia (72,3 de cada 100 000). Los trabajadores del ámbito doméstico y personas sin empleo representaron el 23,1 % de los casos autóctonos. La mayoría de los casos autóctonos ocurrieron entre la 37 ª y la 44 ª semana de 2014 (setiembre y octubre) y casi todas (20 de 21) las ciudades de la prefectura en Guangdong se vieron afectadas. En comparación con la región fuera de la región del Delta del río Pearl, en la región del Delta del río Pearl se registraron casos personas que contrajeron enfermedades a principios de año y la mayoría fueron casos de dengue. Se detectaron los serotipos del virus del dengue 1 (DENV-1), 2 (DENV-2) y 3 (DENV-3), siendo el DENV-1 el predominante (88,4 %).

Conclusiones: La fiebre del dengue es un problema grave de salud pública y continua resurgiendo como una amenaza continuada en la provincia de Guangdong. Existe una necesidad urgente de mejorar el control del dengue, especialmente en poblaciones de alto riesgo y zonas de alto riesgo.

Translated from English version into Spanish by Anna Queralt, through

TRANSLATORS
WITHOUT BORDERS
