# Supplementary figures and images for: Characterizing a large outbreak of dengue fever in Guangdong Province, China
Source: Infect Dis Poverty. 2016 May 3;5:44. doi: 10.1186/s40249-016-0131-z (PMC4853873; doi:10.1186/s40249-016-0131-z)

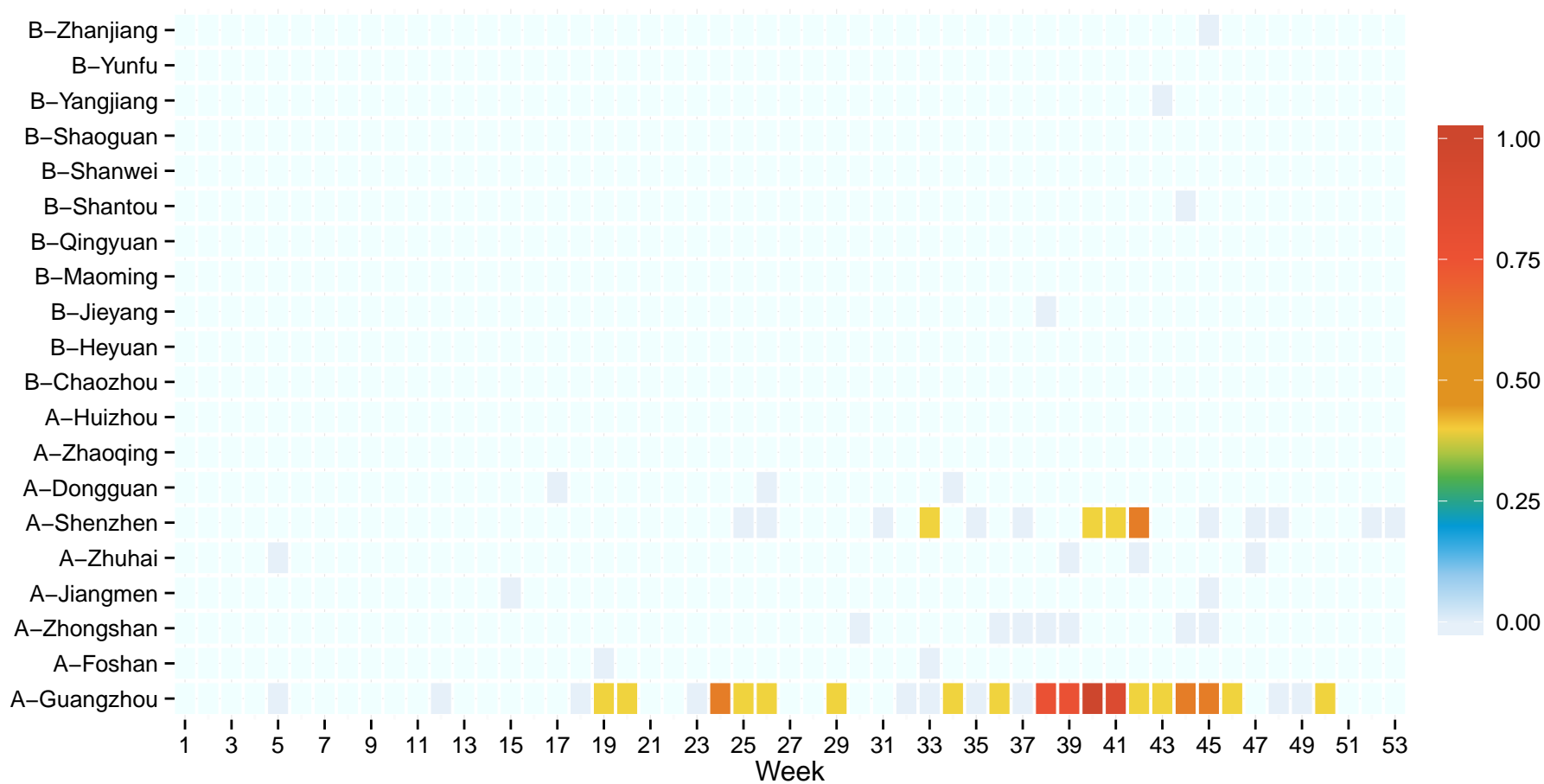

Supplement: Additional file 2: Figure S1. — Heat map of imported dengue cases by city and week in Guangdong Province in 2014. A indicates the Pearl River Delta Region; B labels the non-Pearl River Delta Region. The number of weekly cases was standardized by the number of total cases. (PDF 9 kb) [file 40249_2016_131_MOESM2_ESM.pdf]
